# Modernization of Regression Models to Predict the Number of Deaths from the New Coronavirus Infection

**DOI:** 10.17691/stm2020.12.4.01

**Published:** 2020-08-27

**Authors:** N.N. Karyakin, N.V. Saperkin, A.P. Bavrina, O.V. Drugova, V.I. Klimko, A.S. Blagonravova, О.V. Kovalishena

**Affiliations:** Rector; Privolozhsky Research Medical University, 10/1 Minin and Pozharsky Square, Nizhny Novgorod, 603005, Russia; Associate Professor, Department of Epidemiology, Microbiology and Evidence-Based Medicine; Privolozhsky Research Medical University, 10/1 Minin and Pozharsky Square, Nizhny Novgorod, 603005, Russia; Associate Professor, Department of Medical Physics and Informatics; Privolozhsky Research Medical University, 10/1 Minin and Pozharsky Square, Nizhny Novgorod, 603005, Russia; Associate Professor, Department of Medical Physics and Informatics; Privolozhsky Research Medical University, 10/1 Minin and Pozharsky Square, Nizhny Novgorod, 603005, Russia; Chief Specialist; GC “MedInvestGroup”, 27 Alexander Solzhenitsyn St., Moscow, 109004, Russia; Vice-Rector for Science; Privolozhsky Research Medical University, 10/1 Minin and Pozharsky Square, Nizhny Novgorod, 603005, Russia; Professor, Head of Department of Epidemiology, Microbiology and Evidence-Based Medicine Privolozhsky Research Medical University, 10/1 Minin and Pozharsky Square, Nizhny Novgorod, 603005, Russia

**Keywords:** COVID-19, SARS-CoV-2, coronavirus, predicting the outcome of infection, multivariate regression model, predicting infection-associated mortality.

## Abstract

**The aim of the study** was to modernize the existing prognostic regression models in the context of expanding knowledge about the new coronavirus infection.

**Materials and Methods.:**

The modification of models and the increase in their predictive ability are based on collecting the available data from international and Russian databases. We calculated the traditional descriptive statistics and used the linear regression analysis for modeling. The work was performed using the IBM SPSS Statistics 26.0 and the R 3.6.0 (RStudio) software.

**Results.:**

Manifestations of the COVID-19 epidemic process in several countries were studied; special attention was put to the number of deaths associated with the infection. A significant proportion of severe cases were noted among patients both in Russia and elsewhere. Considering that the disease incidence has reached its peak in China and Italy, we were able to improve the previously published (Sovremennye tehnologii v medicine 2020, Vol. 12, No.2) regression models and to compare their performance. The first modified model is based on the absolute increase in new cases of the infection: its regression coefficient is 0.16 (95% CI 0.137–0.181). In the extended version of the updated model, we additionally considered cases of aggravated COVID-19: the regression coefficients were 0.128 (95% CI 0.103–0.153) for model 2 and 0.053 (95% CI 0.029–0.077) for model 1.1; p=0.0001.

**Conclusion.:**

Based on the most recent data (from January to May 2020) on the incidence of COVID-19 in the world, we have developed more specific versions of the basic and extended regression models of lethal outcomes. The resulting models are optimized and extrapolated to the current epidemiological situation; they will allow us to improve our analytical approach. For that purpose, data collection is currently ongoing.

## Introduction

In view of the continued spread of COVID-19 around the world, intensive work continues in many countries to ensure an adequate response to the individual and social risks associated with this infection. Among other methods, there is a mathematical modeling approach to short-term and long-term prognoses of the epidemiological situation. The COVID-19 incidence pattern has changed since the initial period of the epidemics, and so has our understanding of this process. Among the recently emerged factors, there are the increasing diagnostic capabilities, the impact of socio-restrictive anti-epidemic measures, as well as the changing approaches to registering the cases of COVID-19 associated infection and death [1–6].

Mathematical modeling is widely used for studying the epidemiology of COVID-19. This approach provides information on qualitative and quantitative aspect of the epidemics, the efficacy of anti-epidemic measures, and the needs of the health system in terms of manpower and diagnostic tools for the treatment and prevention.

In January 2020, the number of cases detected in Russia exceeded 20; by May all regions of the Russian Federation had already registered COVID-19. The first deaths from this disease were recorded at the end of March. By today, the infection has spread throughout urban and rural areas and involved various age groups. It is important to note that outbreaks of the infection occurred in medical organizations, as well as among people working on a rotational basis [7, 8]. Therefore, in conditions of the constantly changing reality, especially after some countries had reached a plateau, and with early signs of decline in the number of new cases, the previously proposed mathematical models [9] required modification.

**The aim of the study** was to modernize the earlier developed regression models for predicting COVID-19 associated deaths and incorporate the most recent data on the epidemics.

## Materials and Methods

The present epidemiological study is based on data from open web sources. The search and processing algorithms are described in our previous publication [9].

**Statistical data processing** was carried out using the licensed software IBM SPSS Statistics 26.0 and R 3.6.0 (RStudio) (RVAideMemoire package). The normality of the distribution was tested with the Kolmogorov–Smirnov criterion and the quartile diagrams (quartile graph Q–Q plot). The power of correlation was assessed by the Spearman correlation coefficient and the type of correlation — with the simple and multiple linear regression. The results are presented as M±SD, where M is the mean, SD is the standard deviation, as Me [IQR], where Me is the median, IQR is the interquartile range (Q1–Q3), and as absolute arithmetic and logarithmic values; the percentage values are shown together with their standard deviation (Р±σ_р_%). The level of significance was p≤0.05. In some cases, a 95% confidence interval (CI) was calculated. Comparison of the models was performed using the variance ANOVA tool and the Akaike information criteria (AIC).

## Results and Discussion

**Figure 1:**
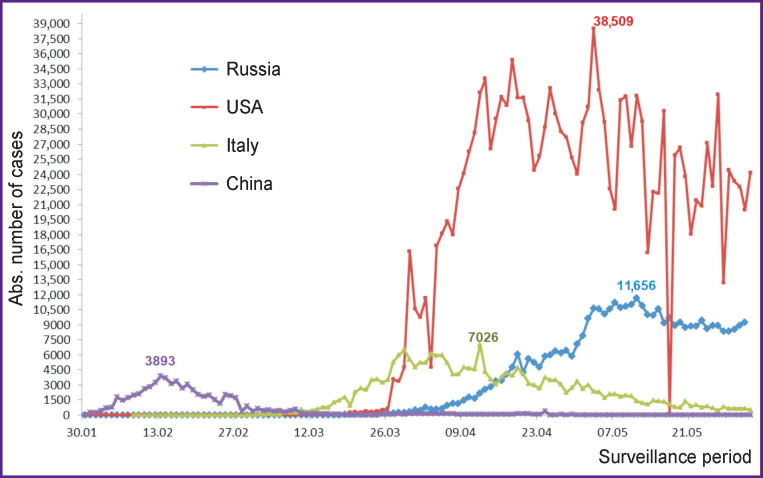
The number of new cases of COVID-19 detected from January to May 2020 (according to officially reported data)

By May 30, 2020, the number of COVID-19 infected people in the world exceeded 5 million, including more than 362 thousand deaths [2]. In Russia, by that time, there were 405,843 laboratory-confirmed cases of infection (with the highest number in Moscow — 180,791, while 9834 cases were registered in the Nizhny Novgorod region). The death rate in Russia is 1.16±0.02% of all confirmed cases [8]. When analyzing the absolute increase in COVID-19 cases (during the period from January 30 to June 1, 2020), a gradual rise in this value in Russia began in April that is later than in Italy and the USA (Figure 1). In addition, the infection rate in the USA significantly exceeded those in other countries throughout the reported period. Notably, in China, there has been a decrease in newly diagnosed cases since the second half of February. The maximum rise in the number of infected people (3893 cases) in China was recorded on February 13, 2020.

**Figure 2:**
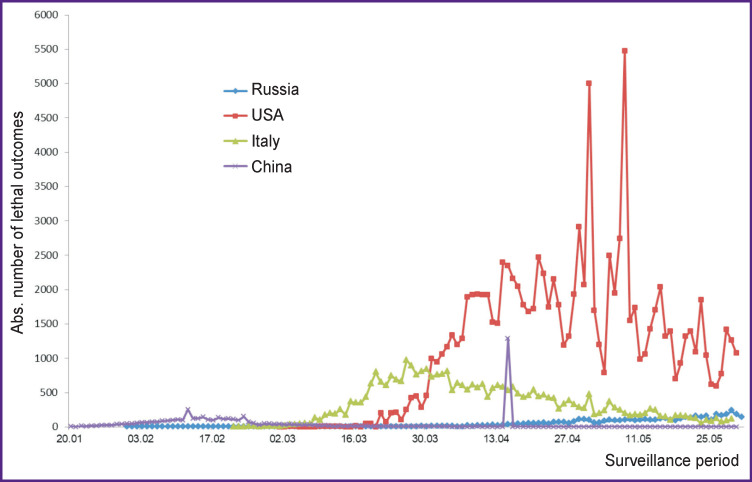
The absolute number of lethal outcomes of COVID-19 recorded from January to May 2020 (according to officially reported data)

When studying the mortality rates, differences between the above countries became evident. Those are about the time when first deaths were reported, the mortality indices, and also the time when the mortality rates began to slow down (Figure 2). Following China, the first deaths were recorded in Italy early enough (February 23, 2020) and the USA (March 3, 2020). Due to the late onset of the COVID-19 epidemic process in Russia, the first deaths began to appear only at the end of March.

In the first third of the pandemic period, fatalities occurred only in China and amounted to ≤260 cases daily. From mid-March, Italy was reporting >900 deaths daily. Notably, from the beginning of April to the present time, most deaths have been observed in the USA: there, the absolute numbers are significantly higher than those in the other three countries. In the USA, sharp fluctuations in the number of deaths have been reported, while the absolute daily increase exceeds 1000 cases, except for isolated days of surveillance.

Below is a statistical description of recent data (incidence and mortality) for COVID-19 in China and Italy in the period after reaching a peak in the number of detected cases (Tables 1 and 2). These data contrast those reported from Russia and the USA at the time of the study.

**Table 1:** Descriptive statistics of the newly diagnosed cases of COVID-19 after reaching a peak incidence

Country	Me [IQR]	Minimum	Maximum	95% CI
China	117 [45–364]	11	894	133–262
Italy	1739 [882–2698]	451	4092	882–2698

**Table 2:** Descriptive statistics of the absolute increase in the number of deaths

China	Me [OQR]	Minimum	Maximum	95% CI
	2 [0–14]	0	150	9–22
Italy	M±SD	Minimum	Maximum	95% CI
	346.1±261.6	0	971	292–400

**Figure 3:**
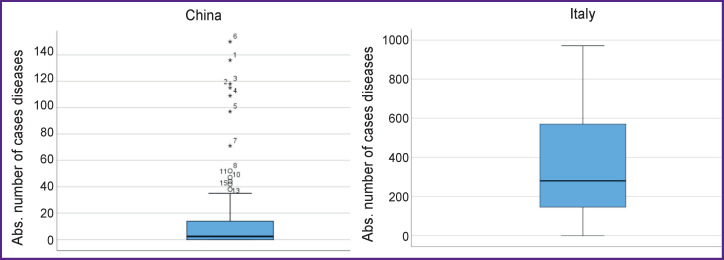
The absolute number of newly recorded COVID-19 cases

Since the data distribution is different from normal, here the median value is used: in the analyzed period (after the peak) the median number of COVID-19 cases in Italy exceeded that for China by more than 14 times (Figure 3). Also noteworthy is the significant difference in new cases between Italy and China.

**Figure 4:**
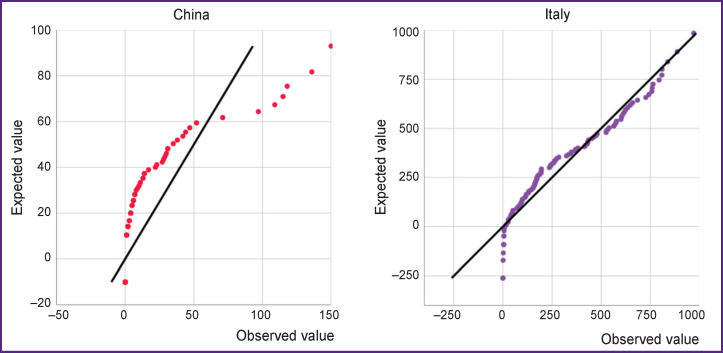
Testing the normality of the data distribution using quartile graphs (Q–Q plot)

Next, the death rate data was tested for normal distribution (Figure 4). When comparing the graphs, a difference in the number of deaths is apparent: the official registry data in the USA fit the formula of the normal distribution, whereas the results for China are distributed asymmetrically. This may be due to a change in the severity of the infection in China resulted in the low number of deaths associated with COVID-19.

After passing the peak incidence, the absolute increase in deaths in China is characterized by a median value of 2 cases [IQR 0–14], while in Italy, the respective value is 346.1±261.6 deaths. In China, the number of deaths did not exceed 150 cases, while in Italy it is approaching 1000.

Our previous study [9] revealed a strong correlation between the occurrence of new COVID-19 cases in different countries. This finding prompted us to start developing predictive models. We built efficient models allowing for calculating the number of COVID-19 associated deaths in countries of interest where the epidemic was approaching its peak. However, after the incidence rate plateaued and began to descend, our earlier developed models needed to be updated so to incorporate the newly reported data.

The new one-dimensional model (model 1.1), based on the similar previous model [9], corresponds to the equation *Y*=*X***·**0.16–1.285 with the determination coefficient R^2^=0.686 (Table 3) and AIC=809.37.

**Table 3:** Characteristics of the new 1.1 model

Constant	Constant value	Standard error	Significance level	95% CI
β_0_	–1.285	2.172	0.556	–5.603…3.034
β_1_	0.16	0.011	0.0001	0.137…0.181

The next version of this regression model (model 1.2) provides for a logarithmic transformation, which makes it possible to assume a linear relationship between the variables (Table 4).

**Table 4:** Characteristics of the new 1.2 model

Constant	Constant value	Standard error	Significance level	95% CI
β_0_	–0.873	0.579	0.137	–2.008…0.262
β_1_	0.749	0.124	0.0001	0.505…0.992

After substituting the coefficients, the equation of linear regression (model 1.2) acquired the form: ln(*Y*)=ln(*X*)**·**0.749–0.873. With the provision for the logarithm operation at zero values, the determination coefficient R^2^ was 0.400, and the AIC criterion — 186.45.

It should be noted that the above models (1.1 and 1.2) can only apply to a limited number of newly detected cases of infection (no more than 30).

Modernization of the previously developed models implied their expansion and inclusion of additional independent variables. As it was shown in the previous publication [9], the inclusion of additional information — the daily absolute increase in severe cases of COVID-19 — led to greater accuracy of the model while maintaining its efficiency. Taking into account the updated calculations, the following model characteristics were obtained (Table 5).

**Table 5:** Characteristics of the new 2 model

Constant	Constant value	Standard error	Significance level	95% CI
β_0_	–5.857	2.2	0.01	–10.311…–1.402
β_1_	0.128	0.012	0.0001	0.103…0.153
β_2_	0.053	0.013	0.0001	0.029…0.077

The new model 2 will take the following form: *Y*=*X*_1_**·**0.057+*X*_2_**·**0.04–9.76.

We also tried to expand the model 2 by using a modified version of these variables (square transformation), but this did not lead to a significant increase in its efficacy.

On the whole, supplementing the model with the number of severe cases not only led to an increase in the determination coefficient R^2^ to 0.741, but also enhanced the accuracy of the model (AIC=793.3). Comparing the models by using ANOVA indicated that the more complex model 2 described the real situation significantly better than did the one-factor model (F-statistic=19.285; p=0.0001).

**Figure 5:**
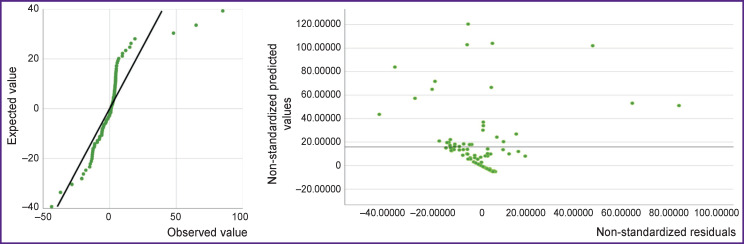
The quartile graph (Q–Q plot) for regression residuals and the distribution of the predicted values by the residuals (for model 2)

The results of testing of the new model 2 for the presence of a linear association between the independent and dependent variables are shown in Figure 5. The linear association is confirmed by the characteristic distribution of non-standardized residuals. There is an even distribution of numerical data, where the dispersion of residuals does not significantly change with an increase in the predicted value, and therefore, the linearity criterion in this regression model is met.

The model was verified by using the number of deaths reported from China on selected dates. The results are shown below.

*Example 1.* After passing the peak of the epidemic in China as of February 28, 2020, we have:

*Y*=0.128**·**435+0.053**·**288–5.857=65 (although in reality there were 44 cases, the order of magnitude is though preserved).

*Example 2.* As of March 5, 2020:

*Y*=0.128**·**29+0.053**·**194–5.857=8 (in reality there were 10 fatal cases on this date but 8 cases were registered after four days).

*Example 3.* As of March 9, 2020:

*Y*=0.128**·**20+0.053**·**317–5.857=13 (on this date, 23 deaths were recorded, but after 4 days, i.e. March 12, 2020, there were 13 deaths).

The data indicate a time gap between the predicted and real values, which will be considered in the further updating of the models.

## Conclusion

By now, several dozens of mathematical models have been developed around the world to predict the dynamics of the COVID-19 epidemic process. Some of the models also aim to determine the efficacy of anti-epidemic and preventive measures and evaluate the needs of health systems. Based on the most recent data on the COVID-19 incidence, we have updated and modernized our regression models (basic and advanced) for predicting the number of deaths. To this end, we carefully studied the pandemic process in Italy and China after these countries had passed the peak of the incidence rates. As a result, the regression coefficients for the intercept and the selected independent variables were updated. Further collection of data is ongoing in the hope to improve the predictive power of these models.
